# Ultrasound assessment of fascial connectivity in the lower limb during maximal cervical flexion: technical aspects and practical application of automatic tracking

**DOI:** 10.1186/s13102-016-0043-z

**Published:** 2016-07-11

**Authors:** Carlos Cruz-Montecinos, Mauricio Cerda, Rodolfo Sanzana-Cuche, Jaime Martín-Martín, Antonio Cuesta-Vargas

**Affiliations:** Department of Physical Therapy, Faculty of Medicine, University of Chile, Santiago, Chile; Laboratory of Biomechanics, San José Hospital, Santiago, Chile; SCIAN-Lab, Programa de Anatomía y Biología del Desarrollo, ICBM, University of Chile, Santiago, Chile; Departamento de Ciencias Morfológicas, Facultad de Ciencia, Universidad San Sebastián, Santiago, Chile; Escuela de Terapia Ocupacional, Facultad de Salud, Deporte y Recreación, Universidad Bernardo O Higgins, Santiago, Chile; Departamento de Fisioterapia, Facultad de Ciencias de la Salud, Instituto Investigacion Biomedica de Málaga (IBIMA), Universidad de Málaga, Andalucia Tech, Cátedra de Fisioterapia y DiscapacidadGrupo de Clinimetria (FE-14), Málaga, Spain; School of Clinical Science, Faculty of Health Sciences at Queensland University Technology, Brisbane, Australia; Facultad de Ciencias de la Salud, Universidad de Málaga, Av. Arquitecto Peñalosa s/n (Teatinos Campus Expansion), 29009 Málaga, Spain

**Keywords:** Fascia, Connective tissue, Myofascial, Tracking motion, Rehabilitation

## Abstract

**Background:**

The fascia provides and transmits forces for connective tissues, thereby regulating human posture and movement. One way to assess the myofascial interaction is a fascia ultrasound recording. Ultrasound can follow fascial displacement either manually or automatically through two-dimensional (2D) method. One possible method is the iterated Lucas-Kanade Pyramid (LKP) algorithm, which is based on automatic pixel tracking during passive movements in 2D fascial displacement assessments. Until now, the accumulated error over time has not been considered, even though it could be crucial for detecting fascial displacement in low amplitude movements.

The aim of this study was to assess displacement of the medial gastrocnemius fascia during cervical spine flexion in a kyphotic posture with the knees extended and ankles at 90°.

**Methods:**

The ultrasound transducer was placed on the extreme dominant belly of the medial gastrocnemius. Displacement was calculated from nine automatically selected tracking points. To determine cervical flexion, an established 2D marker protocol was implemented. Offline pressure sensors were used to synchronize the 2D kinematic data from cervical flexion and deep fascia displacement of the medial gastrocnemius.

**Results:**

Fifteen participants performed the cervical flexion task. The basal tracking error was 0.0211 mm. In 66 % of the subjects, a proximal fascial tissue displacement of the fascia above the basal error (0.076 mm ± 0.006 mm) was measured. Fascia displacement onset during cervical spine flexion was detected over 70 % of the cycle; however, only when detected for more than 80 % of the cycle was displacement considered statistically significant as compared to the first 10 % of the cycle (ANOVA, *p* < 0.05).

**Conclusion:**

By using an automated tracking method, the present analyses suggest statistically significant displacement of deep fascia. Further studies are needed to corroborate and fully understand the mechanisms associated with these results.

## Background

Fascia provides and transmits forces for connective tissues, thereby regulating human posture and movement [1]. Fascial tissue is of great interest to areas such anatomy, biomechanics, and musculoskeletal rehabilitation [2–4]. In regards to rehabilitation research, special interest in the concept of myofascial chains has grown in recent years [4] due to the application of soft tissue manipulation for gaining flexibility and, importantly, decreasing pain [5]. From the perspective of biomechanics, fascia plays important roles in movement restriction and proprioception [2, 6]. This restriction has been observed in fascial connectivity models, such as between the pelvis and deep fascia of the medial gastrocnemius (MG) [2], and has been reinforced by observations in cadaveric models, which suggest posterior fascial connectivity between the thoracolumbar spine, pelvis, and feet [4].

In the context of rehabilitation, various techniques apply the concept of myofascial connectivity to treat musculoskeletal disorders [7], thus highlighting the clinical importance of assessing the degrees of fascial connectivity throughout different regions of the human body. Among the widely used therapies, the fascial release technique assumes that fascial tissue is a continuous layer that spreads throughout the body [7]. Therefore, when fascial tissue is stretched in one area, it can cause tension, restriction, or pain in another area of the body [7]. In terms of general connectivity, while the connection between the thoracolumbar spine, pelvis, and feet is accepted [2, 4], cervical spine flexion should not increase MG tension [8]. However, *in situ* fascial tissue behavior has not been evaluated.

There are two possible pathways through which cervical spine flexion could cause MG fascial displacement. The first is through the posterior lamina of the thoracolumbar fascia, which is one of the main dorsal pathways of force transmission that directly connects to the gluteus maximus and hamstring fascia [9]. The second is through the neurodynamic response of the sciatic nerve [10]. Specifically, Johnson and Chiarelo [11] reported that cervical spine flexion, medial rotation of the hip, and ankle dorsiflexion exert neural mechanical tensions that are transmitted through the spinal cord, spinal dura, and lumbosacral roots to the sciatic nerve. Importantly, peripheral neural tissue has a close relationship with the surrounding fascia by means of the epineurum [10]; so forces transmitted through the neural structures could easily be transferred into fascial tissues. This point is particularly relevant when considering that previous studies have observed increased hamstring flexibility following the release of the suboccipital muscles [12, 13]. While the mechanism associated with this effect is uncertain, it could be due to an interaction between myofascial connections of the hamstring tendons and suboccipital muscle with a neural system that passes through the spinal dura [12–14].

One way to assess the myofascial interaction between the cervical region and lower leg is a fascia ultrasound recording. Ultrasound can follow fascial displacement either manually [15] or automatically through two-dimensional (2D) [2, 16] and three-dimensional (3D) methods [17]. Despite these diverse methods, previous studies have found that during active movements, fascia behaves in a longitudinal and transversal manner, but during passive uniaxial traction, fascia has longitudinal deformations [18]. One possible alternative for tracking of fascia, is the iterated Lucas-Kanade Pyramid (LKP) algorithm, which is based on automatic pixel tracking during passive movements in 2D fascial displacement assessments [2]. This algorithm presents great potential for facilitating dynamic studies of fascia. Nevertheless, the technical aspects derived from evaluation must be determined, since this algorithm has a proven accordance of 0.034 mm with manual tracking [2] and a precision of 0.2 mm in regards to a known distance [19].

Until now, the accumulated error over time has not been considered, even though it could be crucial for detecting fascial displacement in low amplitude movements. This accumulated error occurs as a result of displacement over time, which corresponds to the difference between a set of points and the original positions. If mistakes occur in the tracking between two frames, this will also be reflected as a displacement error that will propagate forward over time, i.e. a basal error. Determining the myofascial interaction between the cervical spine and lower body during postures associated with neural tension would help clarify the possible mechanisms associated with neurodynamic techniques and their interaction with myofascial chains.

Therefore, the main objective of the present study was to determine the basal error of LKP tracking and subsequently assess the MG fascial displacement using an ultrasound with automatized LKP tracking during maximal cervical spine flexion in a maximal kyphotic posture with the knees extended and ankles fixed at 90°.

## Methods

### Participants

Young males were recruited following authorization by and the guidelines of the Ethical Committee of the Northern Metropolitan Health Service of Santiago, Chile. The study complied with the ethical principles of the Declaration of Helsinki.

Before the measurement and the beginning of the study, each participant was given all information about the test and the purpose of the study. Participants had to sign an informed consent form in order to participate in the study. Participation in the study was voluntary and participants could discontinue their involvement at any time. The inclusion criteria for sedentary subjects considered less than 150 minutes per week of moderate physical activity [20]. Subjects were excluded if they presented a history of any musculoskeletal pain in the last six months, pathological conditions of the vertebral column, neurological diseases, respiratory diseases, a systemic rheumatic condition, heritable connective tissue disorders, and/or any previous abdominal surgery.

### Procedure

A 5–10 MHz linear array transducer was used SonoSite TITAN® (Sonosite Inc., Bothell, WA, USA). Ultrasound images were taken at a depth of 39 mm, and video was recorded (Pinnacle Dazzle® DVD Recorder HD, Corel Corp., Ottawa, ON, Canada) at a rate of 30 frames per second and a resolution of 720 × 480 pixels.

**Fig. 1:**
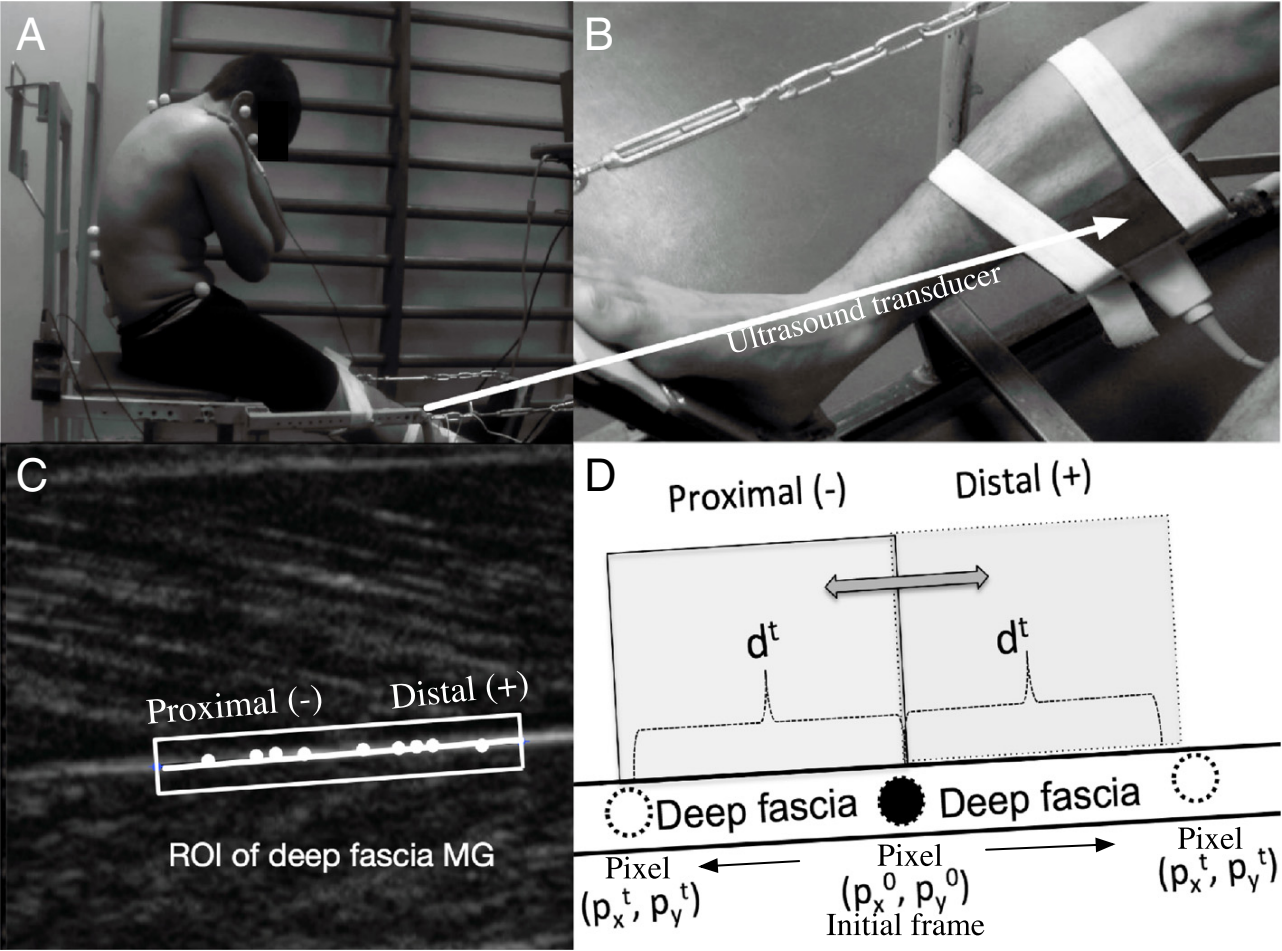
**a** Example setup for kinematic analysis and location of the ultrasound. **b** Colocation of the ultrasound transducer in the medial gastrocnemius (MG). **c** Region of Interest (ROI) and automatic selection of pixels in the deep fascia of the MG. **d** Representation of one pixel in ROI for calculated Euclidean distance (d^t^) between the position in time t (p^t^_x_, p^t^_y_) and the initial frame (p^0^_x_, p^0^_y_)

Video-photogrammetric analysis was performed using the GoPro Hero3 camera (GoPro Inc, San Mateo, CA, USA) at a rate of 60 frames per second and a resolution of 1440 × 1440 pixels. Offline pressure sensors were used to synchronize the 2D kinematic data from cervical flexion and deep fascia displacement of the MG. To determine cervical flexion, an established 2D marker protocol was implemented (Fig. 1a) [21].

The transducer of the ultrasound system was placed on the extreme dominant belly of the MG by attaching a clamping device composed of a thermoplastic polymer and two elastic bands with velcro (Fig. 1b).

All signals and video processing was analyzed with a programing language in Matlab software (MathWorks Inc, Natick, Massachusetts, USA). Lucas–Kanade affine template tracking was used to automatically track the markers of interests [2]. Prior to applying the algorithms, an exploratory consistency assay was performed to evaluate angular variation against the Gold Standard, which is based on the infrared SMART-D 140® recoding system (BTS BioEngineering, Milan, Italy). A 2D angular variation curve of movement determined by a goniometer with three markers was compared at 60 frames per second on a frontal plane with the GoPro. Lin’s concordance correlation coefficient was 0.999, and the average difference between the two methods was 0.360°, with threshold values of 1.0736–1.7946 (95 % confidence interval). The algorithm presented high precision and alignment with the Gold Standard. Finally, the angular variation curves of cervical flexion were processed using a 6 Hz low-pass filter [2].

A previously published methodology for automatic tracking was used to detect deep fascia displacement in the MG (Fig. 1c) [2]. From the automatically selected pixels to be used in tracking, the Euclidean distance (d) was calculated between the positions in time (t), (p^t^_x_, p^t^_y_) and in the initial frame (p^0^_x_, p^0^_y_).

$$ {d}^t=\sqrt{{\left({p}_x^0-{p}_x^t\right)}^2+{\left({p}_y^0-{p}_y^t\right)}^2} $$

Formula 1. Calculation for Euclidean distance (d^t^).

**Fig. 2:**
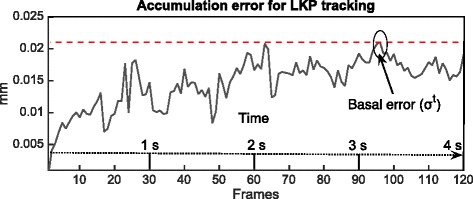
Basal tracking error of Lucas-Kanade Pyramid (LKP). The maximum standard deviation (σ^t^) during the 4 s assay (total of 120 frames)

The initial position on the axis (x) defined displacement in such a way that the distal end was positive and the proximal end negative (Fig. 1d). Since there are no prior reports for a time-related accumulation error for LKP tracking, an additional control experiment was performed in subjects that remained static during an ultrasound video recording of the deep fascia in the MG. This allowed for an evaluation of the basal tracking error in a temporal series. To ensure that subjects remained static, they were asked to suspend their breathing and hold a prone position for 4 s. This length of time was chosen as it was the average time recorded in experimental subjects from the start of movement to the end of the task. The basal tracking error was determined in two healthy men within the set ranges of the experimental groups. From the nine automatically selected tracking points, the Euclidean distance (d^t^) was calculated. There was an average difference of 0.0063 mm in d^t^ (0.0664 pixels) between two consecutive frames, and the maximum standard deviation (max) (σ^t^) was 0.0211 mm (0.2230 pixels) during the 4 s assay (total of 120 frames). This final error was defined as the basal tracking error (Fig. 2)

$$ \max \left({\sigma}^t\right)= \max \Big(\sqrt{{\displaystyle \sum_{k=1}^9}{\left({d}_k^t-{\displaystyle \sum_{p=1}^9}{d}_p^t*\frac{1}{9}\ \right)}^2*\frac{1}{9}\Big)} $$

Formula 2. Calculation for the basal tracking error.

To establish a reflex muscle contraction in the MG, the amplitude of muscle activity was recorded by surface electromyography at a sampling rate of 1000 Hz (Artoficio®, EMG VIII, Santiago, Chile). The skin was cleaned and the electrodes were positioned according to SENIAM recommendations [22]. To obtain a signal, a pass-band (20–450 Hz; Butterworth fourth order) and a band-stop filter (50 Hz; Butterworth fourth order) were used. Following this, the root mean square was applied with a 250 ms window. To assess electrical activity during the task, basal muscular activity was determined based on the average between a second before initiating the task and a second at the end of cervical spine flexion.

**Fig. 3:**
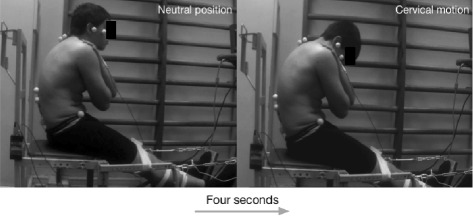
Active cervical flexion starting in a neutral position and moving until maximum flexion

The task was practiced for 5 min prior to measurements, starting with the neck in a neutral position, as achieved by maintaining horizontal sight and keeping the other corporal segments relaxed and without any movement, and moving towards cervical spine flexion. Each subject was asked to perform each task three times. Each execution was standardized with a metronome so that the subject would finish each execution within 4 s. Maximal cervical flexion was performed in a sitting position with the lumbar and dorsal spine locked in maximal kyphosis and the knee fixed at full extension by a strap across the patella, which was adjusted as tightly as possible without causing pain or irritation for the subject. Ankles were locked at 90° by resting the foot on a static platform. Cervical flexion started from a neutral position to one of maximal flexion (Fig. 3). The task was considered successful if movement was only recorded for the cervical spine and, if this condition was not met, the test was nulled.

### Data management and statistical analysis

Preliminary values were obtained from a pilot study in six subjects. The values of fascia displacement in the first 10 % of the cycle and those at the end of the cervical spine flexion were considered for sample size analysis. Since a statistical power of 90 %, an alpha of 5 %, and a loss rate 30 % were used to detect differences equal to or above 0.037 mm and with a standard deviation of 0.027 mm, the calculated sample size for the experimental condition was a minimum of 10 subjects.

Final average displacement was determined such that it would be above the basal LKP tracking error, thus determining the existence of deep fascia displacement of the MG. All data were analyzed with the SPSS for Windows 22.0 software package (IBM Corp, Armonk, New York, USA). A value of *p* < 0.05 was considered statistically significant. To determine normal distribution, the Shapiro-Wilk test was used. All data were normally distributed. To assess average MG fascial displacement, ANOVA testing was used, and readings from every 10 % of the cycle were compared against the first 10 % of the cycle.

## Results

All participants were male and dominantly right-handed in the inferior train. Fifteen participants were chosen to perform the cervical flexion task. The characteristics of the participants were as follows: an age of 23 ± 2.3 years, height of 1.75 ± 0.06 m, and body mass index (kg/m^2^) of 24.21 ± 1.31.

**Fig. 4:**
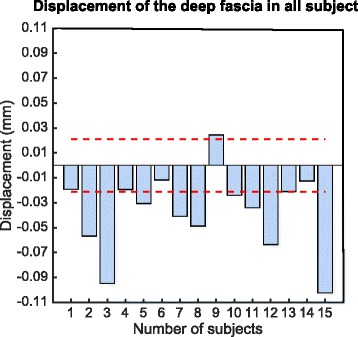
Total deep fascia displacement of the medial gastrocnemius in all subjects. Red dotted line indicates the basal tracking error

**Fig. 5:**
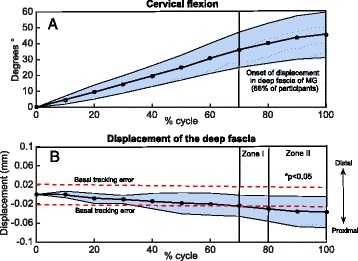
**a** Range of motion of cervical spine flexion. **b** Medial gastrocnemius (MG) deep fascia displacement. Zone I shows the onset of MG deep fascia displacement. Zone II shows the cycle percentage where fascia displacement was greater than 10 % of the movement cycle with *p* < 0.05

Proximal fascial tissue displacement was observed in 66 % of the participants. Total displacement after subtracting the basal error was 0.076 ± 0.006 mm (Fig. 4). The onset of fascia displacement during cervical spine flexion was detected over 70 % of the cycle; however, only when detected for more than 80 % of the cycle, the displacement was significantly greater than the first 10 % of the cycle (ANOVA, *p* < 0.05) (Fig. 5). The onset of fascia displacement occurred at 36° of cervical spine flexion, equivalent to 76 % of maximal cervical spine flexion. The MG electromyography increased 2.6 % in regards to the basal value, but this was not statistically significant (*p* = 0.452).

## Discussion

Deep fascia displacement of the MG was observed in 66 % of the subjects over 76 % of the final range of motion in cervical spine flexion. This displacement, which was above the basal error, showed an average proximal displacement of 0.076 mm. The same LKP tracking method was previously used in another report, obtaining distal fascia displacement of 1.50 mm in the MG during pelvic anteversion with the knees blocked in full extension and the ankles at 90° [2]. Even though displacement in the present study was slight, it is relatable to previous studies that have detected minimal displacement of the sciatic nerve at the thigh during cervical spine flexion in a sitting position [23].

Since peripheral neural tissue has a close relationship with the surrounding fascia through the epineurium, force transmission might not only be through fascial tissue, but also through neural tissue [24]. During cervical flexion, the facial pathway may be transmitted through the posterior lamina of the thoracolumbar fascia, which is one of the main dorsal pathways of force transmission that directly connects to the gluteus maximus and hamstring fascia [9]. The neural pathway, which involves the spinal cord, spinal dura, and lumbosacral roots, creates tension in the sciatic nerve with different types of joint movements, including cervical spine flexion [10, 11]. In the hamstring, the fascial tissue and sciatic nerve have a connection through the epineurium.

A previous study showed different amounts of nerve excursion with different types of neural mobilization techniques, showing that slider mobilization creates greater longitudinal nerve displacement than tension or single joint mobilization [23]. In clinical practice, the interaction between suboccipital musculature inhibition and hamstring flexibility has been empirically evaluated [12, 13]. This effect can be explained by a connection of the hamstring tendons with the suboccipital muscle through a neural system that passes through the spinal dura, part of the posterior myofascial chain [12–14].

Until now, only one study has reported on the effects of the myofascial release technique with manual follow-up and manual tracking in ultrasound videos [15]. Regarding the tracking of fascia, this methodology can be used in future research to more fully explore aspects related to the transmission of myofascial force in different musculoskeletal and neurological pathological conditions. Specifically in manual therapy, there is great potential in assessing the effects of myofascial release techniques locally or over a distance by using automatized tracking methods.

The proximal displacement of the MG reported in this study might be a result of interactions between the neural and fascial tissues, especially considering that the cervical spine flexion test used in this study resembles the Slump test [25]. From a practical point of view, the results reported in this study provide new perspectives on the ultrasound assessment of fascial tissue, particularly for determining slight displacements and in providing new in vivo perspectives to apply and reinforce the concept of myofascial connectivity over a distance with a neurodynamic approach.

Nevertheless, further analyses of these results are needed, especially when considering that a recent study found cervical flexion to produce a non-significant sciatic nerve excursion during the slump test, as compared with upright-sitting and slump-sitting [26]. These differing results may be due to the use of a standardized 45 cm diameter ball in the present study for maintaining a slump position. When maximal cervical flexion in a maximal kyphotic posture is assessed, different results can be produced as compared to a standardized posture for all subjects. This would consequently produce different myofascial and nerve tensions.

Nevertheless, further studies are needed to compare different automatic tracking methods in fascial displacement assessments. For example, neural tissue assessments have used the cross correlation method that is based on the follow-up of a region of interest between two consecutive images [23, 26]. The precision between the cross correlation method and LKP tracking for neural and fascial tissue follow-up remains to be evaluated.

### Study limitations

Regarding the limitations of this study, the slight facial displacement observed during cervical spine flexion was only evaluated in one position in the spine and hip. To fully determine the possible influence of spine position in myofascial transmission, further research that assesses different degrees of kyphosis is necessary. Furthermore, the methodology only considered the assessment of fascia in a relaxed position. Due to this, it was not possible to assess how different levels of trunk activation may induce fascia displacement. Moreover, this study focused only on men, and future research should consider comparative studies between genders, particularly regarding gender hormone differences that influence muscle flexibility and joint laxity [27, 28].

Moreover, future studies should additionally consider a simultaneous assessment of neural and fascial tissues and determine the anthropometric influence of facial displacement. Finally, while the present results show a certain tendency towards MG fascia displacement during cervical spine flexion, more studies are needed to corroborate this finding.

## Conclusion

In conclusion, by using an automated tracking method, the present analyses evidence statistically significant displacement of deep fascia. These results suggest myofascial connectivity between the cervical spine and lower limbs. Further studies are needed to understand the mechanisms associated with these results.

## Abbreviations

2D, two-dimensional; 3D, three-dimensional; LKP, Lucas-Kanade Pyramid; MG, medial gastrocnemius
